# Spatial association of biosocial and economic factors with reproductive women obesity in urban India

**DOI:** 10.1371/journal.pone.0319580

**Published:** 2025-03-18

**Authors:** Priya Das, Subhadeep Saha, Tanu Das, Partha Das, Tamal Basu Roy

**Affiliations:** Department of Geography, Raiganj University, Uttar Dinajpur, West Bengal, India; Wuhan University, CHINA

## Abstract

Obesity creates several health complications among the urban women from reproductive age group. So far it is most ignored public health concern particularly in Indian context. The study aims to focus on the identification of cluster of districts with obese urban women and its spatial association with selected spatial determining explanatory factors.This study utilized secondary data obtained from the fifth round of the National Family Health Survey (NFHS-5), 2019-2021.The study performed spatial cluster of districts through univariate Moran’s *I* and its association with selected determining factors through bivariate Local Indicator of Spatial Association (BiLISA). Geographically Weighted Regression (GWR) was applied to measure the magnitude of independent factors over the space affecting the prevalence of outome of urban obese women.The spatial autocorrelation value of obesity among the urban women was found 0.429, depicting the moderate concentration of obesity coverage among the urban women over the districts of India. The results of bivariate LISA revealed that the highest bivariate Moran’ I value among all the predictors were identified for those women who had caesarean delivery (I = 0.274), followed by non-poor population (I = 0.208). The adjusted R^2^ value evidenced by the GWR model was 0.727 indicated that the employed explanatory variables was explaining about 73% for making influence on the prevalence of obesity among urban women of reproductive age group across the districts of India. This study recommends for an urgent need of interventions of the target areas focusing predominantly the urban women belonging from higher socio-economic status.

## Introduction

Both in developed and as well as in developing countries, obesity is now one of the most prevalent but neglected public health problem. An abnormal or excessive fat accumulation in the body that could be deleterious for human health, is referred to as obesity. Body Mass Index (BMI), which is again calculated by using weight, height and age, is primarily used to measure obesity. According to World Health Organization (WHO), an individual having elevated BMI which is over 30 is considered as obese [1].Obesity typically develops when a person can’t burn as much energy as they consume, starts to pile up and gain excessive weight [2]. Over the last few decades, there have been seen a tremendous increases in cases of obesity worldwide [3].As per the recent estimates provided by WHO, globally one in eight people are living with the problem of obesity. Currently, 43% of adults are overweight (43% of men and 44% of women) while 16% are obese (11% of men and 15% of women [4]. By forecasting, it can be predicted that if the current trends continue, 18% of the men and 21% of the women globally would be obese by 2025 [5].

The obesity-related health issues are referred to as complex metabolic disorder which is sometimes also termed as lifestyle diseases include cardiovascular disease, diabetes, arthritis, hypertension, high cholesterol, certain type of cancers, polycystic ovarian disease, alzheimer disease, asthma, liver steatosis etc [6,7]. Obesity is more prevalent among women than men in most of the countries [8]. Particularly, women of reproductive age group (15-49 years) are more sufferer to obesity due to different factors such as pregnancy, use of different hormone contraceptive pills, menopause, infertility treatment, hormonal issue etc [9]. Obesity among reproductive age group is associated with different pregnancy complications such as gestational diabetes, gestational hypertension, pre-eclampsia or eclampsia, postpartum hemorrhage and adverse birth outcome such as preterm birth and ever perinatal death of newborn [10,11].

The increased prevalence of obesity has been imputed to different factors such as globalization and socio-economic development which have continuously alterted the human behaviours [12].Obesity might be a major common problem of most of the developed countries but nowadays, the outbreak of this epidemic is also becoming rampant in some of the developing countries [13]. In developing countries like India where there hunger and malnutrition still exists, however have witnessed an increasing burden of obesity prevalence [14,15]. Researchers already documented that from preceding three decades, socio-demographic changes, economic development, and different cultural and environmental changes have been become very impressive in developing countries, thereby leading to a continuous curtailment in underweight prevalence with a simultaneous increasing figure in overweight and obesity prevalence. Previously, different studies attributed that type of physical activity, diet pattern, more consumption of fast food and fatty food, sedentary lifestyle, stressful life and environmental pollution were some of the responsible factors affecting the increasing prevalence of obesity [16–21]. Interestingly, urban environment and increasing urbanization itself doesn’t directly cause obesity, but it exacerbate the underlying causes related to develop obesity and may contribute to the likelihood and severity of such incidents [22].

**Fig 1:**
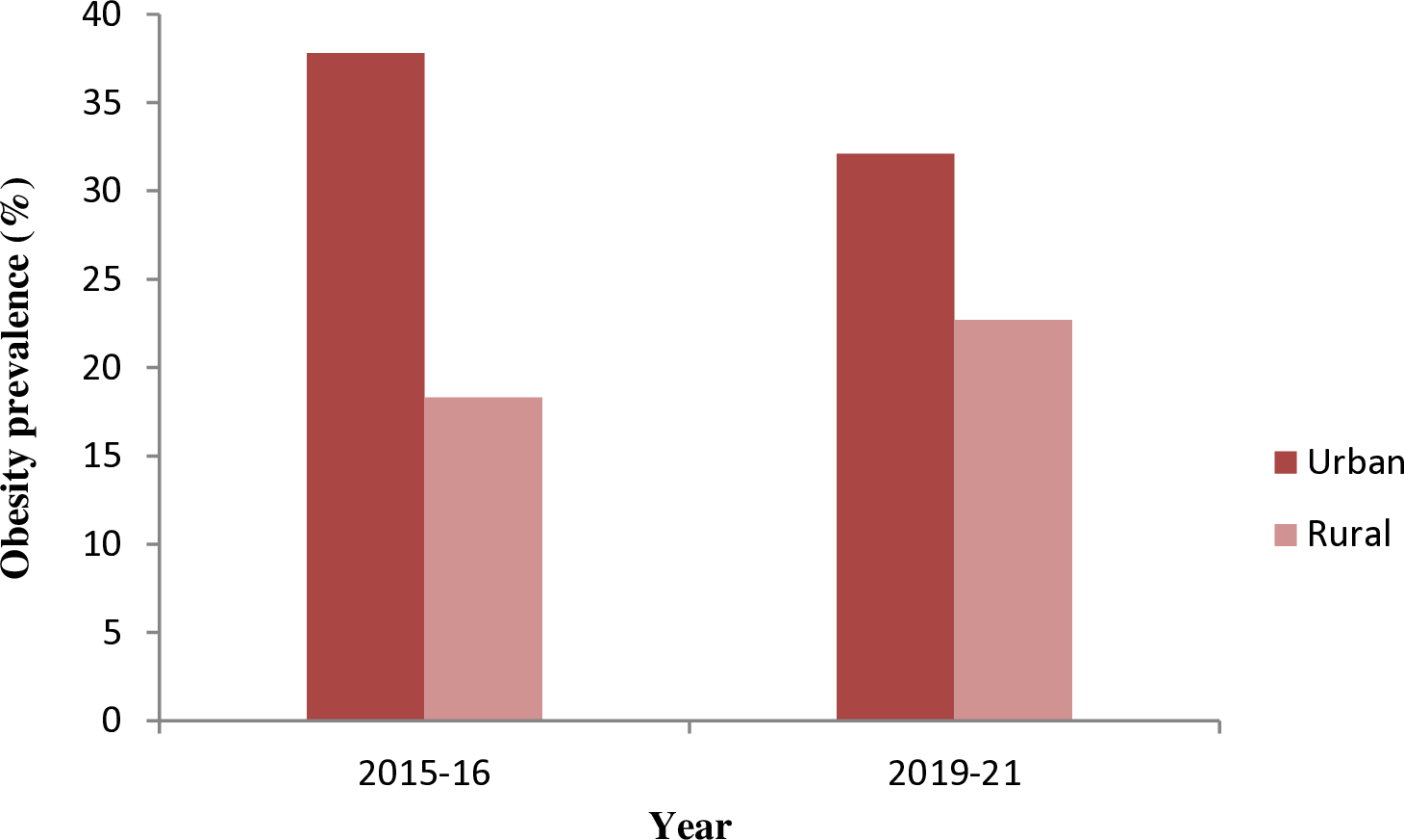
Prevalence of obesity among women of reproductive age group (15-49 years) by place of residence, NFHS-4 (2015-16) and NFHS-5 (2019-21).

The obesity prevalence rates in India are increasing alarmingly. Currently India ranks third globally in terms of obesity, just behind the US and China from the list of top 10 countries with highest number of obese individuals. Recent estimation showed that 70% of India’s urban population is found obese [23]. Although previouly enormous literature highlighted about the different risk factors of obesity among women in reproductive age group in different country context but there are relatively few studies showing current scenario of India. Even, there is almost no study dealing only with the urban women at national level (Fig 1). However, it is an emeging health issue in recent times, required urgent interventions, but studies are found relatively scant. This research study very clearly reflects that the factors which is most common in urban environment are significantly associated with the obesity prevalence. This study shows regional variation of obesity prevalence, its spatial pattern and its association with the undelying employed factors across the districts of India. Additionally, the Geographically Weighted Regression (GWR) was applied to explore how the selected prdeictors influence the obesity prevalence from place to place. This study may further help to identify the target areas to assign relevant stakeholders and suggest a shift from adopting national-level policies to localized strategies focusing on certain target areas which may be more cost-effective but can definitely be considered as the most useful measures for reducing the incidence of obesity and its associated adverse consequences among the urban women of reproductive age group across India.

## Database and methodology

### Data source

This study relied on secondary data found from the most recent (fifth) round of the National Family Health Survey (NFHS-5), carried out in India by the International Institute of Population Sciences (IIPS), Mumbai from 17 June, 2019 to 30 April, 2021. This nationally representative, large-scale cross-sectional demographic health survey data provided socio-economic, demographic and health related information not only at the household level but also at the individual levels. This survey also yielded the much needed estimates on fertility, mortality, maternal, child and adult health, women and child nutrition etc. at the national, state and as well as district levels. The entire survey for data collection has been framed using a stratified two-stage sampling design, encircling a vast number of populations, approximately 636,699 households, 724,115 women aged 15-49 years and 101,839 men aged 15-54 years distributed across the 707 districts spanning 28 states and 8 union territories. On an average, 940 households were surveyed in each district. District is the unit of analysis of this study. NFHS-5 also prepared a district level data file based on some emergent demographic and socio-economic indicators. The district level estimates are publicly available from https://rchiips.org/nfhs/districtfactsheet_NFHS-5.shtml whereas the unit level data for public use are available from the online repository of the Demographic and Health Survey (DHS) https://dhsprogram.com/data/ through a request.

### Study participants

NFHS-5 provided information about 724,115 women in reproductive age group (15-49 years). Since the present study was dealt with only the urban women so, at first, the respondents from rural areas were excluded from the data file. Only those women residing in urban areas were limited for this current study. After that, the present study considered only those women of reproductive age group who have given complete data on the anthropometric outcome. Furthermore, women who were currently pregnant during the survey period were excluded as their weight would not be the representative. So, after justifying all inclusion and exclusion criterion, the present research study was restricted to170,111 women aged 15-49 years who lived in urban areas.

### Outcome variable

The main outcome variable for this present study was obesity prevalence among reproductive aged urban women, which was here measured through the Body Mass Index (BMI) of the women. Body Mass Index (BMI) is basically a measure of body fat, calculated as weight in kilogram divided by height in meters squared (expressed as kg/m^2^) that applied to adult men and women. Following World Health Organization’s (WHO) criteria of classifying BMI, women who have BMI equal to 30 or higher (BMI ≥ 30) were considered as obese and those having BMI ≤ 30 were not obese. The study’s outcome was used as the proportion of obese women across the 707 districts in India.

### Exposure variables

This study employed a set of fivedominant factors associated with the obesity prevalence among women, assuming that these variables might influence obesity prevalence pattern and its variations among urban women of reproductive age group in India. These five variables were district wise proportion of women who have completed more than 10 years of schooling, non-poor population indicating the proportion of the population belonged to the higher wealth quintile, proportion of women who ate fast food daily (fried food or aerated drinks), the proportion of women who delivered by caesarean section and also the proportion of women who ever got their pregnancy terminated. The proportion of all the selected variables was calculated from the unit level data of NFHS-5 (2019-21). District wise attributes are entried with the concerned spatial unit (district).

Below Table 1 provided a detailed explanation of dependent and independent variables selected for this study.

**Table 1:** Description of the study variables.

Variable name	Description	Source
**Dependent variable**		
1. Obesity prevalence (%)	Percentage of urban women of reproductive age group having BMI ≥ 30 in a district	
**Independent variable**		
1. Higher educated women (%)	Percentage of women with more than 10 years of schooling in a district	
2. Non-poor population (%)	percentage of women belonging to higher wealth quintile in a district	NFHS-5 (2019-21)
3. Eating fast food (%)	Percentage of women who frequently consume fast food (fried food or aerated drinks) on daily basis in a district	
4. Ever had caesarean delivery (%)	Percentage of women who ever had caesarean delivery in a district	
5. Ever had terminated pregnancy (%)	Percentage of women who ever had terminated pregnancy in a district	

### Methodology

The entire study results were obtained through applying univariate Moran’s *I,* bivariate Local Indicator of Spatial Association (BiLISA) and geograhically weighted regression technique systematically. The objective has ben fulfilled to get a district wise overview of clustering of urban obese women and its bivariate association with selected underlying predictors which were later incorporated in final geographically weighted regression (GWR) model. The final footstep of GWR was carried out to measure the magnitude of effect of specific predictor at district level to get through the outcome issue.

Before conducting the Geographically Weighted Regression (GWR) model, we have checked multicollinerarity by computing Variance Inflation Factor (VIF). If the VIF score was found 10 or more, then the explanatory variables are expected to have multicollinerarity between them. So, the VIF value of 10 or more was considered as unacceptable.


$$VIF=\frac{1}{1-R^{2}}$$

Where, R^2^ represents the coefficient of determination for regressing th ith explanatory variable

### Univariate Moran’s *I* -

Moran’s *I* is one of the popular and frequently used methods to measure spatial autocorrelation at the neighbourhood level around a specific spatial location. Spatial autocorrelation typically quantifies the extent to which data points show similarity or dissimilarity to their neighbouring spatial units. It also determines the extent of spatial non-stationarity and serving as a means to detect and delineate clustering, randomness or dispersed neighbourhood spatial relations. The formula used to calculate univariate Moran’s *I* is as follows-


Univariate Moran' s I=nSO×∑i∑jWijXi−X¯Xj−X¯∑iXi−X¯)2

Where,*x*_*i*_and *x*_*j*_ represents the proportion of obesity prevalence in district *i* and *j* respectively; $\bar{X}$ is the mean of the proportion of obesity; n is the number of spatial units; W_ij_ is standarized spatial weight matrix between observation *i* and *j*based on contiguity of each spatial units and S_o_is the aggregation of all spatial weights (S_o_=∑_*i*_ ∑ _*j*_W_*ij*_).The spatial weight matrix explains spatial proximity between two potential districts. In this study, in order to perform Moran’s I, we have used Geo-da software where the spatial weight matrix (W_ij_) was calculated using queen contiguity method. The queen contiguity method is the most easiest method for knowing the neighbourhood influence by polygon features where the analysing spatial units (here districts) share a boundary or corner with the nighboring units.

The value of Moran’s I ranges in between -1 to + 1, where + 1 indicates perfect clustering and -1 value indicates perfect dispersion. Positive moran’s I value indicates positive spatial autocorrelation which means points having similar attribute values (either High-High or Low-Low) are clustered together whereas negative spatial autocorrelation denotes that points having dissimilar attribute values are clustered together. Moran’s I value closer to 0 or 0 denotes that there is no significant spatial autocorrelation, suggesting a random or uniform spatial pattern across the study area.

### Bivariate Local Indicator of Spatial Association (BiLISA)-

Bivariate Moran’s I along with Bivariate Local Indicator of Spatial Association (BiLISA) technique examines the clustering pattern, identifies ouliersand investigates the relationship between an outcome variable and a predictor variable of a specific geographic location.In this study, the BiLISA technique was utilized in the way where obesity prevalence among urban women of reproductive age group was taken as the observation variable which was represented on the y-axis and chosen explanatory variables namely women’s higher education, non-poor population, eating fast food, having caesarean delivery and having terminated pregnancy was opted as lagged value variable which was represented on the x-axis. Here, the bivariate LISA technique yielded five cluster maps, their associated significance maps and Moran’s I scatter plots for each sets of variables.

The equation for calculating bivariate Moran’s I is-


$$\text{Bivariate Moran}\;'\text{s }I=\frac{n}{S_{O}}\times \frac{\sum_{i}\sum_{j}W_{ij}\left(X_{i}-\bar{X}\right)\left(Y_{j}-\bar{Y}\right)}{\sum_{i}\left(Y_{i}-\bar{Y}\right){)}^{2}}$$

Where, x and y represens the interest variables; $\bar{X}$ is the mean of x and $\bar{Y}$ is the mean of y; n is the number of spatial units; Wij is standarized spatial weight matrix between observation *i* and *j*and S_o_is the aggregation of all spatial weights (S_o_=∑_*i*_ ∑ _*j*_W_*ij*_).

Bivariate LISA is estimated by-


$$BivariateLISA\left(I_{i}\right)=\frac{n.\left(X_{i}-\bar{X}\right)}{\sum_{i}{\left(Y_{i}-\bar{Y}\right)}^{2}}\sum_{j}W_{ij}{\left(Y_{i}-\bar{Y}\right)}^{2}$$

The bivariate LISA method produces a map displaying two types of statistically significant clusters (High-High and Low-Low), two types of outliers (High-Low and Low-High) and one class indicating non-significant areas.The ‘High-High’ clusters exhibit spatial units where the interest variable has high values and the area is also surrounded by high values in neighbouring areas. The ‘Low-Low’ cluster comprises geographical areas having low values of interest variable, is encompassed by similar low value neighbours areas. The ‘High-High’ clusters are commonly reffered to as “hotspots” due to their higher prevalence whereas owing to lower prevalence the ‘Low-Low’ clustes are recognised as “coldspots”. Moreover, two types of spatial outliers are also detected by this technique: one corresponds to high prevalence geographic areas surrounded by low prevalence neighbouring areas, denoted as ‘High-Low’ and the another corresponds to low prevalence geographic areas surrounded by low prevalence neighbouring areas, termed as ‘Low-Low’.

**Geographically Weighted Regression (GWR)-**

In this study, Geographical Weighted Regression (GWR) was applied to understand the non-stationary effect of the selected variables at the local-level. GWR generally provides an effective methodology for examining spatially varying regression models and explores how predictor variables affect the outcome variable from place to place.It estimates results at the local level rather than global level by providing different regression coefficients for each region separately which indicates spatial heterogenity of the outcome.TheKernel-based weighting approach creates the local spatial weights in GWR model. The equation for calculating GWR is expressed as follows-


$$y_{i}=\beta_{0}\left(u_{i},v_{i}\right)+\beta_{1}(u_{i},v_{i})x_{1i}+\beta_{2}(u_{i},v_{i})x_{2i}+\ldots \ldots .+\beta_{k}(u_{i},v_{i})x_{ki}+{€}_{i}$$

Where, y_i_ is the obesity prevalence among urban women of reproductive age group at district i; *x*_1i_, *x*_2i_,…,*x*_ki_ are predictor variables at district i;

β_0_ (u_i_,v_i_) is the intercept term specific to location (u_i_,v_i_);

$\beta_{1}($ u_i_,v_i_)*x*_1i_ + $\beta_{2}($ u_i_,v_i_)*x*_2i_ + ……. + $\beta_{k}($ u_i_,v_i_)*x*_ki_ are location-specific regression coefficient for each predictor at (u_i_,v_i_);

ɛ_i_ is the error term.

GWR is theoretically consistent with the acknowledgment of spatial heterogeneity in relationships, recognizing that the factors influencing the prevalence of urban women’s obesity can differ significantly across India’s different geographical regions. More specifically, it can be stated that this concept acknowledges the fact that there are regional variations in social, economic and health aspects influencing the prevalence of obesity among urban women. Methodologically, by providing different regression coefficients for each region separately, GWR offers localized insights which may help policymakers to create specific actions, targeted interventions and policies that can fulfill the requirements of the areas with higher prevalence of particular challenges.

Univariate Moran’s *I* and Bivariate Local Indicator of Spatial Association (BiLISA) were carried out using GeoDa (version-1.22) software. For obtaining the results of Geographical Weighted Regression (GWR),the latest version of ArcGIS software (version-10.8.2) was used.

## Results

### Spatial scenario of obesity prevalence among urban women in India

**Fig 2:**
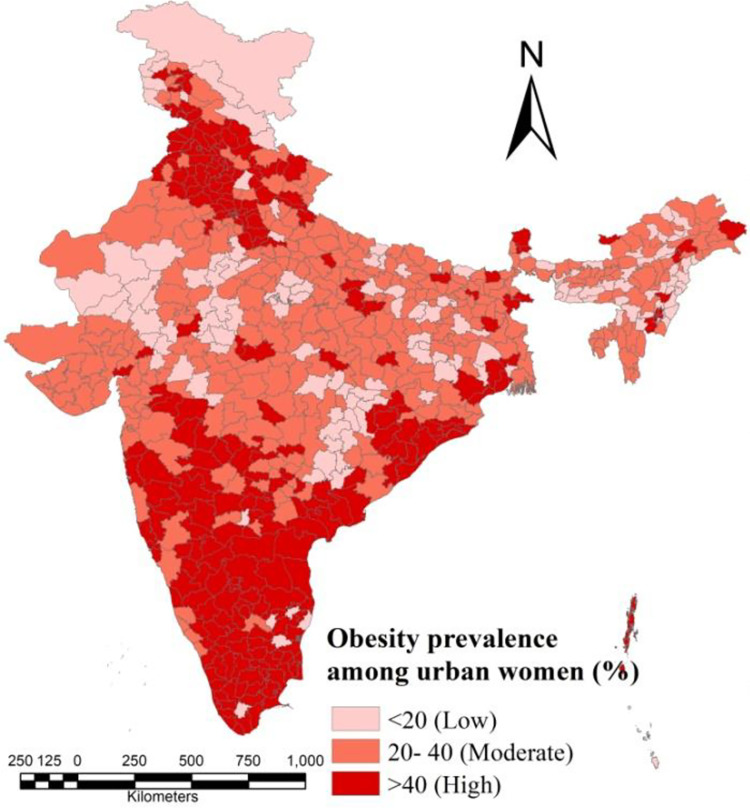
District wise percentage distribution of obesity among urban women in India.

Obesity prevalence among urban women in India is obviously a matter of concern in recent times; however, still neglected issue that is rarely addressed locally. Regarding the percentage prevalence of obesity among urban women, there exists a clear and consistent difference throughout the states and districts. As per the data provided by NFHS-5, the prevalence of obesiy among urban women is higher among the districts of Punjab, Haryana, Odisha, Andhra Pradesh, Telengana, Karnataka, Kerala, Tamilnadu, parts of Maharashtra. These states are mainly located in north-western and southern parts of India (Fig 2).

**Fig 3:**
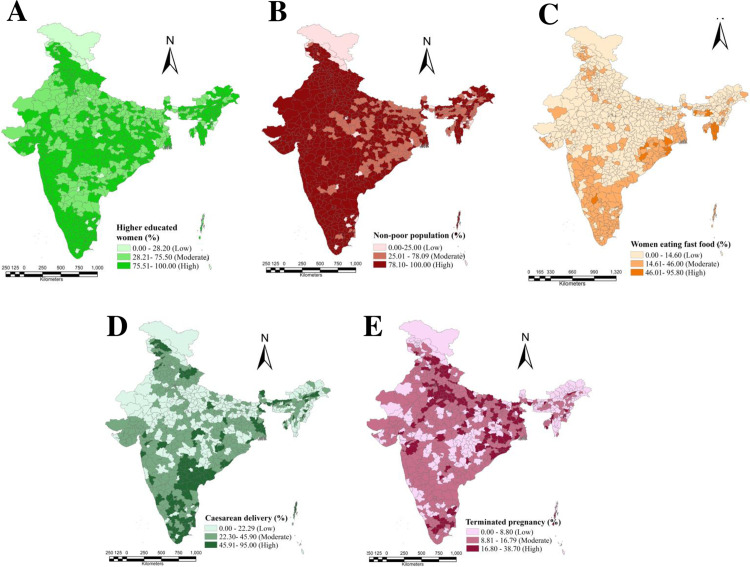
District wise spatial distribution of the selected explanatory factors: (A) Women with higher education; (B) Non-poor women population; (C) Women eating fast food; (D) Women had caesarean delivery; (E) Women had terminated pregnancy.

Fig 3 visualizes the spatial distribution of different selected explanatory variables affecting the obesity prevalence among urban women over the 707 districts of India.

### Results of Spatial autocorrelation analysis

**Fig 4:**
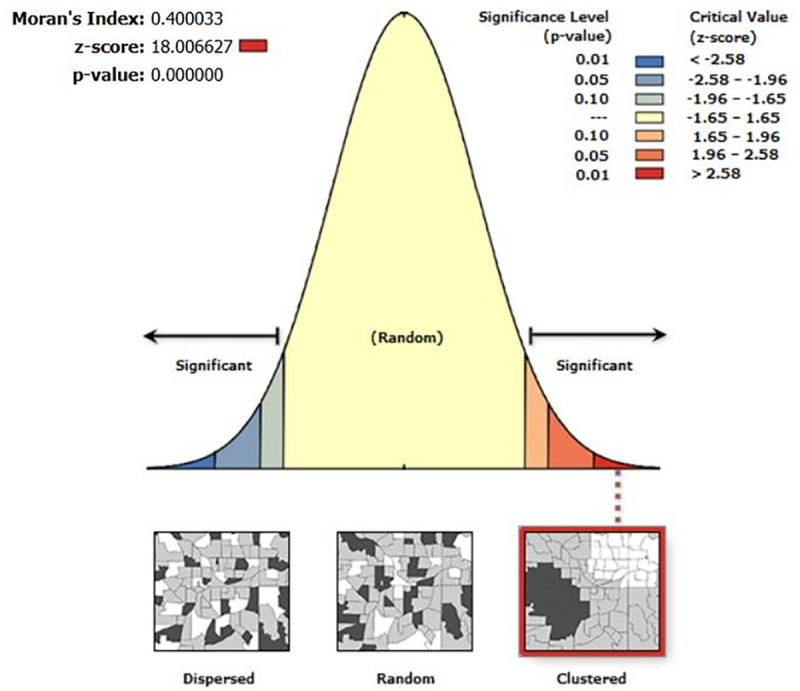
Graphical representation of the result of spatial autocorrelation of urban women’s obesity prevalence in India.

Fig 4 depicted the graphical representation of the result of spatial autocorrelation of urban women’s obesity prevalence in India. On the basis of the results of spatial autocorrelation, the distribution of urban women’s obesity was found clustered in India (Moran’s I value = 0.400, Z-score = 18.006, p-value = 0.000).

**Fig 5:**
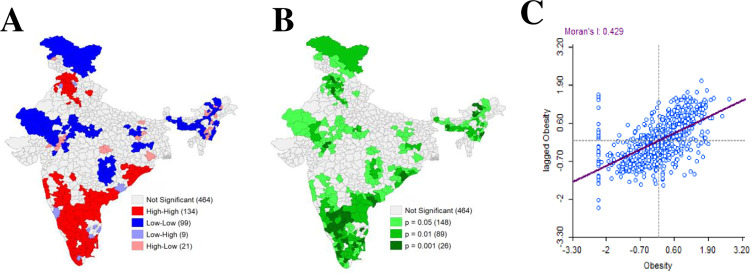
Univariate LISA map of obesity among urban women in India: (A) Univariate cluster map, (B) associated significance map and (C) Moran’s I scatter plot.

Fig 5 represented univariate cluster map, associated significance map and Moran’s I scatter plot to get through the linear association of prevalence of urban women’s obesity of specific district and lagged district. The cluster map clearly reflected that high obesity prevalence among urban women of reproductive age group was mainly concentrated in the north-western and almost entire southern parts of India, comprising 134 districts across India. A significant High-High clustering was found in these 134 spatial units which were commonly referred to as hotspot areas of urban women’s obesity. The High-High cluster was formed due to similar high-value influence in adjacent areas. On the other hand, 99 coldspots concerning Low-Low clustering were observed in the western, north-eastern and some of the eastern parts of India where lower concentration of urban women’s obesity was noticed.

Here, the scatter plot visualized the relationship between the values of urban women’s obesity and the lagged values of urban women’s obesity. The scatter plot having four quadrants showed different types of spatial clusters. Concentration was mainly found in the upper-right corner exhibiting High-High clusters and in the lower left corner was comprised with Low-Low clusters. Some spatial outliers were also examined in the lower right (High-Low) and upper left (Low-High) of the quadrants.

### Results of bivariate local indicator of spatial association

**Fig 6:**
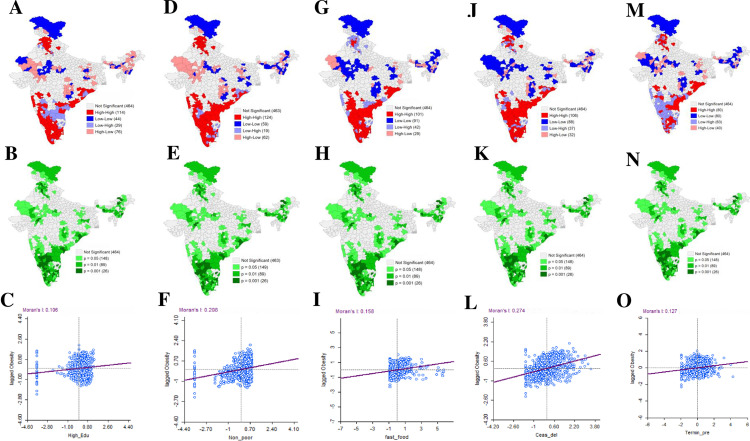
Bivariate Local Indicators of Spatial Association (BiLISA) map, associated significance map and Moran’s I scatter plot representing the spatial clustering pattern based on bivariate relationship between outcome variable and selected explanatory variable: (A, B, C)- Urban women’s obesity and Higher education; (D, E, F) - Urban women’s obesity and Non-poor population; (G, H, I) - Urban women’s obesity and Eating fast food; (J, K, L) - Urban women’s obesity and Caesarean delivery; (M, N, O) - Urban women’s obesity and Terminated pregnancy.

Fig 6 delineated the bivariate LISA cluster maps for obesity among urban women in reproductive age group by different predictor variables along with their associated significance map and bivariate Moran’s I statistics for urban women’s obesity against each predictor (Table 2).The outcome showed that all the bivariate Moran’ I value and their associated Z-score appeared with greater significance levels. Results revealed that the highest bivariate Moran’ I value among all the predictors were identified for those women who had caesarean delivery which was 0.274 at significance level < 0.001, followed by non-poor population (I = 0.208). Results further expressed that the spatial autocorrelation between urban women’s obesity and its relationship with eating fast food was 0.158 whereas that with women having pregnancy termination was 0.127 and that of women with higher education was 0.106.Each and every Moran’ I value witnessed a positive spatial autocorrelation with urban women’s obesity.

**Table 2:** Bivariate Moran’s I value, Z-score, significance level and spatial pattern.

Dependent Variable	Independent variable	Moran’s I	Z-score	Significance level	Spatial pattern
	Higher educated women	0.106	5.995	<0.001	Clustered
	Non-poor population	0.208	12.246	<0.001	Clustered
Urban women’s obesity prevalence	Eating fast food	0.158	19.341	<0.001	Clustered
	Ever had caesarean delivery	0.274	17.907	<0.001	Clustered
	Ever had terminated pregnancy	0.127	12.792	<0.001	Clustered

The outcome of Bivariate LISA cluster maps (Fig 6) depicted that, in case of urban women’s obesity and women with higher education, the High-High clusters were observed in 114 districts of 707 districts across the India, mostly from southern part of the country except Andhra Pradesh and partly from northern India. About 16% of all the districts of India were designated as hotspot regions of urban women’s obesity with relation to women with higher education while the Low-Low clusters were observed for 44 districts. The outcome of BiLISA cluster maps also reflected that a higher concentration of non-poor population acted as an accelerating factor of obesity prevalence among urban women. For non-poor population and urban women’s obesity, 17.5% area of all over India was identified as hotspot areas. In contrast, parts of north-eastern and eastern states, some districts of central India witnessed Low-Low clustering pattern, commonly termed as coldspot regions of the same. Likewise, a cluster of 101 districts of 707 across India (around 14%) were recognized as hotspot regions of urban women’s obesity in respect to women eating fast food. A total of 106 districts (around 15%) across India emerged spatially concentrated as High-High, revealing a greater percentage of those women delivered by caesarean section exposed towards being obese. Similarly, in case of those urban women who ever had their pregnancy terminated, High-High clustering was visualized in 80 spatial units. The hotspot regions of urban women’s obesity against all the predictors were mostly found from southern and north-western parts of India whereas the coldspot regions were more or less located in the western, northern, central and north-eastern states of India.

### Results of Variance Inflation Factor (VIF)

Before conducting the Geographically Weighted Regression (GWR) model, we have computed the Variance Inflation Factor (VIF) to check multicollinerarity among the chosen explanatory variables. The mean VIF value was found 1.03 which indicated that the analysis was free from multicollinerarity that the study can proceed for conducting the Geographically Weighted Regression (GWR) with the selected explanatory variables.

### Results of Geographically Weighted Regression (GWR)

Geographically Weighted Regression (GWR) is a very effective technique for modeling the dynamic spatial patterns in the dataset by considering the varying nature and intrinsic relationship of the selected variables at local neighborhood level. In this current research, the Geographical Weighted Regression was run in order to investigate the non-stationary spatial influence on urban women’s obesity prevalence at district level. The R^2^ value of GWR model generally indicates how well the employed independent variable can predict the dependent variable. Here, the adjusted R^2^ value of GWR was found 0.727 which indicates that the employed explanatory variables was explaining about 73% for making influence on the prevalence of obesity among urban women of reproductive age group across the districts of India. The model further suggested that there was only 27% of variability that might be unexplained by this model at local neighborhood level where some of the other unknown predictors which was not included in this study might influence the obesity prevalence.

**Fig 7:**
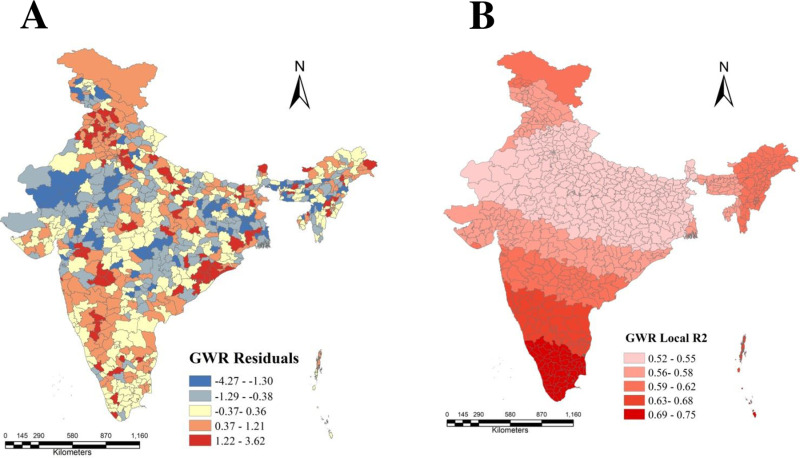
Spatial distribution of (A) residuals and (B) local R2 of GWR model for urban women’s obesity prevalence associated with the significant covariates across the districts of India.

The estimated local R^2^ found from GWR were showed by map in Fig 7. The map visualized that the increasing local R^2^ was mainly observed in the southern parts of India covering Kerala, Tamilnadu, parts of Karnataka and some parts of Andhra Pradesh state. The districts with higher R^2^ values exhibited with greater concentration of urban women’s obesity prevalence by the explanatory variables. A thin stretch of southern India and parts of north-east India showed moderate local R^2^ that is 0.59 to 0.62, indicating improved explanatory power of data. The figure moreover indicated that this model did not fit well for the districts of central India, suggesting that the lower obesity prevalence among urban women of this area might be directed by the influence of the employed covariates.

**Fig 8:**
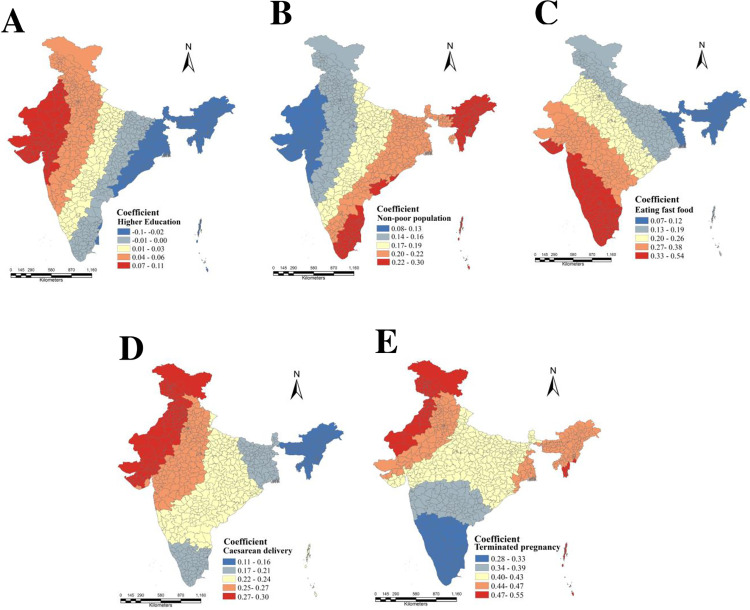
Coefficient map of five selected explanatory factors estimating urban women obesity across the districts of India: (A) Urban women’s obesity and Higher education; (B) - Urban women’s obesity and Non-poor population; (C) - Urban women’s obesity and Eating fast food; (D) - Urban women’s obesity and Caesarean delivery; (E) - Urban women’s obesity and Terminated pregnancy.

In addition, the GWR model estimated local coefficients of five selected explanatory variables which were visualized in Fig 8 (A, B, C, D, and E). This figure outlined some observable spatial patterns of the study area. The geographical pattern of parameter estimates between urban women’s obesity and employed explanatory variables were illustrated by these maps. The spatial association between urban women’s obesity and women with higher education showed higher positive coefficient values, concentrated in western and northern parts of India. Generally, positive beta coefficient indicates accelerating one factor (independent) can regulate the other factor (dependent). Here, the positive coefficient suggested that the higher percentage of educated urban women stimulated the tendency to become obese among them locally. Conversely, a weak spatial association of this case was observed in the eastern and north-eastern India having negative co-efficient value. The spatial distribution of the local coefficient between non-poor population and urban women’s obesity differed from the patterns discussed for earlier predictor. In this case, the higher beta coefficient covered the southern and north-eastern part of India, indicating obesity prevalence among urban women of this area was regulated by a higher proportion of non-poor population prevailing there. The coefficient map for the predictor eating fast food revealed that proportion of women who had fast food eating habits had a strong relation with obesity prevalence among urban women at the local scale, where more consumption of fast food had more chances to exacerbate women’s obesity. It was evident from the Fig 8C that the urban women of south India more consumed fast food like fried food and aerated drinks, accelerated obesity prevalence among them. The beta coefficient of caesarean delivery and pregnancy termination (Fig 8D & 8E) had found a positive association with obesity prevalence among urban women of reproductive age group in the western and north-western parts of India. Here, the women of reproductive age group who ever had caesarean delivery or ever got their pregnancy terminated showed more likelihood to become obese. The outcome for both the predictors showed more or less similar spatial concentration across the districts of India.

## Discussion

This study highlighted the spatial pattern and distribution of the burden of obesity among urban women of reproductive age group and moreover investigated the underlying spatial association between the same outcome and five dominant explanatory variables such as women with higher education, non-poor population, eating fast food, ever had caesarean delivery and ever had terminated pregnancy among those urban women of 15-49 years across the 707 districts in India. Estimation showed that about 33% of urban women of reproductive age group of India had the problem of obesity according to the latest survey of National Family Health Survey (NFHS-5). The spatial pattern of obesity prevalence among urban women in India visualized that the urban women of southern India are more obese in comparison to other parts of India. This could be demonstrated as South India always has a greater performance in terms of education, socio-economic development, GDP growth and human development [24].The study found that the contribution of women’s higher education in any area is significantly associated with urban women’s obesity prevalence in India. The female literacy rates are much higher in most of the states of south India. Most of the women are highly educated there [25]. Higher educated women generally involve themselves in different Governmental and non-governmental organizations, private firm for their personal income which again helps them to maintain higher socio-economic status [26,27].They generally conduct sedentary and unhealthy life practices, frequently exposed to unhealthy diet pattern, less strenuous physical activity etc in their daily life. Educated women remains most of the time busy at their work, mostly go their workplace by their own private car and even never do any household work, always seeking help from their helping hand or subordinates, almost not involved in any physical activities. Their daily unhealthy lifestyle is continuously making them vulnerable to become obese [28].

The result of the study also revealed that non-poor population of any area has a strong significant association with prevailing obesity among them in India. In southern India, most of the women hail from above the poverty line in comparison to other states of India. Southern states are much ahead in terms of economic growth and better income. Nowadays, better income and with economic capabilities, the food habit of every family is continuously changing; higher consumption of sugar-contained food and beverages, higher consumption of meat and dairy products, more consumption of processed foods may contribute to develop obesity among women of economically rich families [29–31]. Some women belonging from high economic background consume excessive amount of alcohol daily for maintaining social status, some of them suffering from job stress or personal stress which also might affect them from suffering obesity [32,33]. Not only that, less strenuous physical activities of economically well-established families due to always having helping hands at home or working in office involving no physical labor or always travelling in personal car may also make feasible reasons for becoming obese [34]. These might be some possible reasons of high concentration of obesity prevalence in most of the southern states of India [35].

The study findings also pointed out that urban women’s obesity prevalence in India is spatially clustered in southern India and some north-eastern states with relation to consumption of fast food daily. In this area, the prevalence of obesity is greater in a large extent than national average. Even in some states like Kerala, Tamilnadu, Andhra Pradesh, Karnataka, Punjab and Delhi, the issue is more prevalent and needs to take immediate actions. The study found obesity increases with increasing intake of different fast food. Earlier studies have already explained that from last few years’ consumption of fast food eaten away from home has increased alarmingly [17]. Generally fast foods are contained high in fat (saturated fat and trans fat), calories and cholesterol which are very much harmful for every human, may lead to develop obesity like disease too quickly [36,37].

The major findings of this study provided some empirical evidences on the scenario of women’s obesity among urban women of reproductive age group across the districts of India which may have momentous public health implications. We all know reproductive span that is 15 to 49 years is the foundation of every woman’s health in later life [38].This study identified some reproductive issues such as having experience of caesarean delivery and pregnancy termination which are closely linked to the prevalence of obesity among those urban women of reproductive age group [39]. This finding was supported by a previous study conducted in India where it was reported that the women who ever had any caesarean births were 57% more likely and the women who ever experienced terminated pregnancy had 20% more risks of having obesity [40]. Pregnancy termination which includes either miscarriage or abortion contributes the reproductive age group women at elevated risks of developing obesity due to various hormonal functions. After miscarriage, hormone fluctuations can have a drastic impact on a woman’s weight and metabolism, resulting in the development of hypothyroidism [41,42]. After C-section or miscarriage, the women also begin to consume a calorie-rich diet to suffice the nutritional needs of their body, when their intake of food exceeds the amount of food required, definitely adds up to the fat leading to unwanted excessive weight gains and consequently they starts to become obese [43]. Usually women after caesarean delivery or any kind of pregnancy termination, take excessive rest for months, even don’t do their routine household works. So, there is actually no scope of burning the excessive amount of calories that they have taken and at times they loses energy and the high amount of calories starts to pile up in their body which increases their BMI and develops obesity [36].

The study faced few limitations. As the data being cross-sectional in nature, there is absence of evidence regarding temporal relationship due to which some of the factors that were detected might not be causally related with the obesity prevalence. Besides, NFHS-5 did not provide any dataset related to physical activity, sleeping patterns, walking hours etc. which might have direct influence on obesity prevalence. In NFHS-5, the responses regarding Body Mass Index (BMI) which was used to detect obesity was self-reported which might create potential biases to the results.

Due to data unavailability, we could not conduct longitudinal studies regarding this issue. But future research is very much necessary to establish the causal relationship between obesity prevalence and various biological, social, and economic factors. Long-term follow-up and repeated measures are very much needed to longitudinally investigate the effect of different biosocial and economic factors on obesity prevalence among urban women aged 15–49 in India.

## Conclusion

The present study comprehensively explored the spatial distribution of obesity prevalence among urban women of reproductive age group in India, emphasizing on significant regional cluster of disparities. The study visualized a greater prevalence of urban women’s obesity in southern parts of India. Obesity is a complex disease, mostly prevalent in recent times and it may increase the risk of developing other health problems too. The research findings of this study will provide valuable insights to policy makers for recommending policies and targeted interventions at the local level mainly focusing the urban women belonging from higher socio-economic status. The focus group identification and adopting the appropriate footstep to minimize the obesity may reduce the burden of BMI anomaly in certain geographic location in India. Limiting unhealthy foods, adequate walking, regular exercise, work, balanced nutrition, managing stress etc. are some essential steps to reduce the incidence of obesity at local and also in global level.

By reiterating the significance of policy interventions for dealing with the long-term effects and health outcomes of obesity among urban women in India, multi-sectoral collaboration and persistent efforts is imperative to encourage healthy lifestyles and lessen obesity prevalence. Government can take initiatives by promoting healthier food environment such as by taxation on unhealthy foods, restricting market of unhealthy foods, subsidizing healthy foods, by conducting different educational campaigns and programs etc. Last but not the least, this study suggests a shift from adopting national-level policies to localized strategies by local health agencies focusing on certain geographic clusters which may be more cost-effective but can definitely be considered as the most useful measures for minimizing the incidence of obesity among urban women from reproductive age group in India.
